# An International Survey of Health Care Services Available to Patients With Tourette Syndrome

**DOI:** 10.3389/fpsyt.2021.621874

**Published:** 2021-02-26

**Authors:** Tracy Bhikram, Rana Elmaghraby, Elia Abi-Jaoude, Paul Sandor

**Affiliations:** ^1^Department of Psychiatry, University Health Network and University of Toronto, Toronto, ON, Canada; ^2^Youthdale Treatment Centre, Toronto, ON, Canada; ^3^Department of Psychiatry, The Hospital for Sick Children, University of Toronto, Toronto, ON, Canada

**Keywords:** Tourette syndrome, pharmacotherapy, health services, health care delivery, comorbidity, clinician survey

## Abstract

**Objective:** Tourette syndrome (TS) is a neuropsychiatric disorder that is highly associated with several comorbidities. Given the complex and multifaceted nature of TS, the condition is managed by a wide variety of practitioners in different disciplines. The goal of this study was to investigate health service delivery and care practices by clinicians who see TS patients across different geographic settings internationally.

**Methods:** A comprehensive questionnaire was developed to assess clinical care resources for patients with TS and was sent to clinicians in Canada (CA), the United States (US), Europe (EU), and the United Kingdom (UK). Responses were compared quantitatively between geographic regions.

**Results:** The majority of respondents, regardless of region, reported that fewer than 40% of their case-load are patients with tics. The accessibility of TS services varied among regions, as indicated by differences in wait times, telemedicine offerings, comorbidity management and the availability of behavioral therapies. First-line pharmacotherapy preferences varied among physicians in different geographical regions with CA respondents preferring alpha-2-adrenergic agonists and respondents from the UK and EU preferring dopamine receptor antagonists.

**Discussion:** The results suggest that there is a scarcity of specialized TS clinics, potentially making access to services challenging, especially for patients newly diagnosed with TS. Differences in regional pharmacotherapeutic preferences are reflected in various published treatment guidelines in EU and North America. The lack of dedicated specialists and telemedicine availability, coupled with differences in comorbidity management, highlight the need for interprofessional care and holistic management to improve health care delivery to patients with TS.

## Introduction

Tourette syndrome (TS) is a developmental neuropsychiatric disorder characterized by motor and vocal tics that is estimated to affect 0.52–0.77% of children and 0.05% of adults (1, 2). Approximately 90% of patients with TS have at least one other psychiatric diagnosis; these comorbidities contribute to the wide spectrum of functional impairment and associated distress seen in patients with TS (3).

The most appropriate treatment for TS varies depending on the clinical presentation. When active treatment of tics is required, comprehensive behavioral intervention for tics (CBIT) has been empirically validated by well-designed studies (4, 5). However, its usefulness is limited by a lack of well-trained practitioners, high costs, and by patients' ability to devote the time and effort it requires (6); pharmacological treatment is therefore required for many patients with TS. The dopamine receptor antagonists (DRAs) are often effective but can be associated with potential serious adverse effects (7). Thus, given their more favorable side effect profile, the alpha-2 adrenergic receptor agonists (A2AAs) clonidine and guanfacine are commonly used in the treatment of TS (8), though their efficacy is lower than that of the DRAs, and their utility is further limited by their own side effects (9). Other pharmacological agents have only limited and weak evidence to support their use.

Adults and children with TS have lower quality of life than the general population (10), as evidenced by lower scores on measures of psychological health and adaptive functioning (11–13), as well as lower socioeconomic statuses (14). Lower quality of life measurements in TS samples (15) suggest the need for more health care services in this population. Indeed, compared to the general population, patients with TS are more likely to report greater health care needs, are more likely to need medication, and use more medical, mental health and educational services (16, 17).

While it has been demonstrated that patients with TS require more health care services, little is known about the different models of care available to TS patients. Previous research has shown that TS clinical populations are similar across different countries and continents (18), but it is unclear what clinical resources and therapeutic practices are available to patients with TS and how these are distributed within (19–22) and across different geographical regions. In order to better understand the resources available to patients with TS, we designed and distributed a survey for healthcare practitioners who work with TS patients. The survey included comprehensive questions that sought out details regarding availability and accessibility of health care services to TS patients from different regions.

## Methods

### Survey Development

A comprehensive questionnaire was developed to assess the clinical care approaches and resources that are available to TS patients internationally. The electronic survey was compiled through SurveyMonkey and reviewed by experienced neurodevelopmental psychiatrists (EAJ and PS) who provided constructive feedback and survey improvements. The revised survey was then distributed for pilot testing amongst the members of the Tourette Syndrome Neurodevelopment Clinic at the Toronto Western Hospital. After further revisions based on feedback from the piloting, the finalized survey consisted of 30 questions, most of which were multiple-choice, but also included rank-order and open-ended questions. The survey questions had two themes, the first one focused on the clinic and setting in which the respondent practices, while the second related specifically to the personal practice of the respondent.

### Study Participants

Lists of health care providers that manage TS, including physicians and non-physicians, were obtained through international TS organizations: Tourette Syndrome Foundation of Canada (now called Tourette Canada), Tourette Syndrome Association (now called Tourette Association of America), Tourettes Action, and Tourette Syndrome Portal. The lists included contact numbers for clinicians in English speaking Canada (CA), the United States of America (US), the United Kingdom (UK), and Europe (EU). Clinicians were then contacted by phone to collect email addresses and request permission to distribute the survey link. The survey link was distributed by e-mail via SurveyMonkey to the clinicians who expressed interest in completing the study. Email reminders were sent 1 week apart, for 3 weeks, if the surveys were not completed. The survey took place from December 2014 to May 2015, with the bulk of responses collected in December and January.

### Data Analysis

The survey data was collected via SurveyMonkey and exported to SPSS (Statistical Package for the Social Sciences). Survey responses were compared between and within geographical regions. Qualitative and quantitative approaches were used to provide detailed descriptions of the responses to the survey questions. Descriptive analyses were repeated on the subset of respondents who indicated that >60% of their patients have tics, regardless of geographical location, to examine the responses among “tic specialists.”

## Results

The survey was distributed to 484 clinicians; 123 clinicians responded, for an overall response rate of 25%. An overwhelming majority of respondents indicated that they practiced in an urban setting (93%), more specifically, at teaching hospitals (51%), private clinics/practices (24%) and community hospitals/clinics (14%). The catchment area for most of the respondents ranged from several hundred thousand to several million people. Respondents included mostly physicians (41%), psychologists (33%) and nurses (6.5%). Of the physician respondents, 46% identified as psychiatrists, 32% as neurologists, 16% as neuropsychiatrists, and 6% as pediatricians.

### Regional Health Service Delivery

The respondents were grouped based on their geographical location, so that the overall sample was divided into 4 regional samples: n(CA) = 21, n(US)=33, n(UK)=28 and n(EU)=41. The response rate was 22%, 37%, 47%, and 17% for each of the regions respectively.

**Figure 1:**
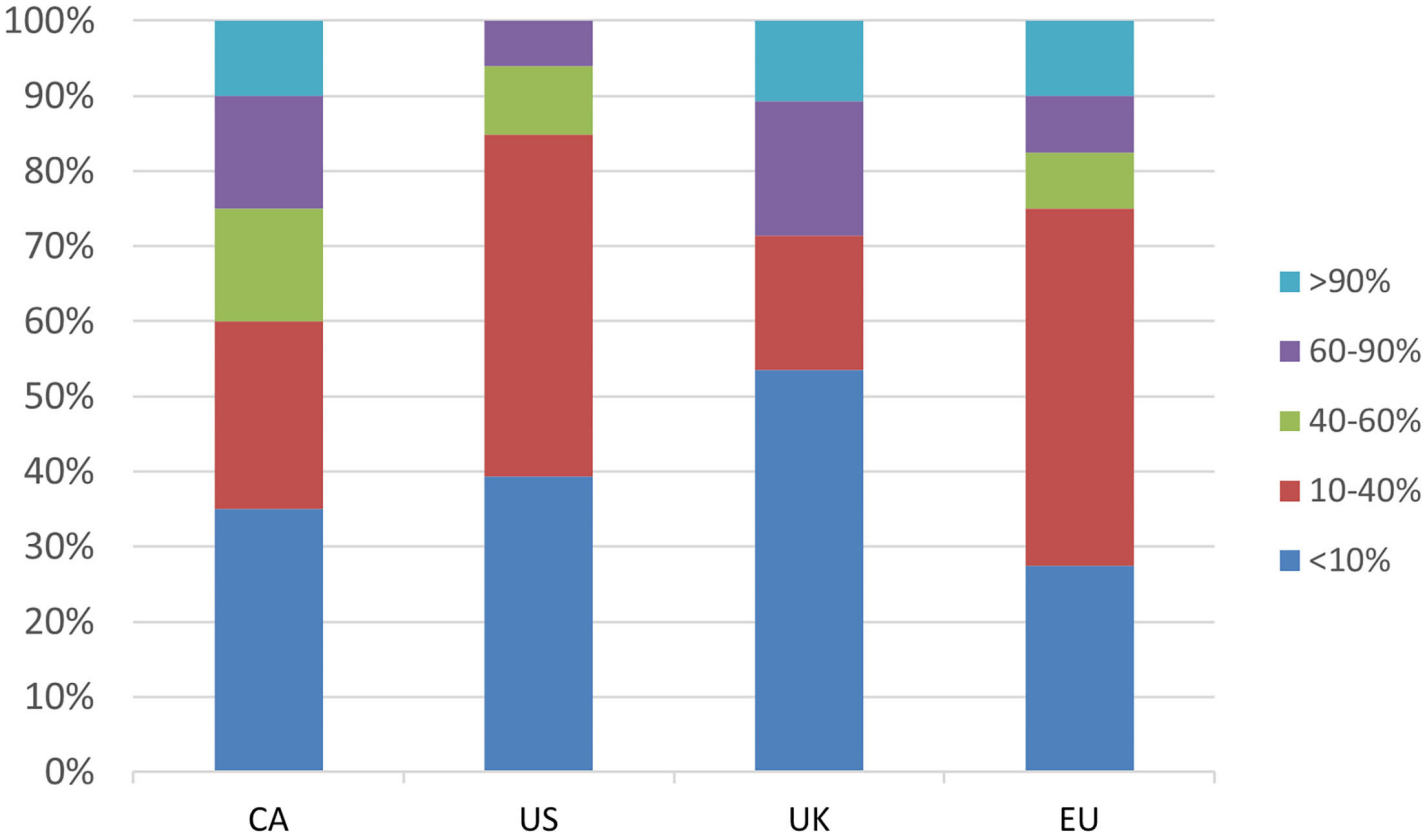
Response break-down for the question: “What percentage of your patients have tics?”

Only a minority of respondents (ranging from 6% in the US to 29% in the UK) reported having a clinic population in which most patients (>60%) have a tic disorder (Figure 1). The majority of respondents in the UK (52%), CA (52%) and US (79%) indicated that they see 0–2 new TS patients/month, whereas the most frequent response in the EU (32% of respondents) was 3–6 new patients per month, and 15% saw more than 15 new TS patients/month (Supplementary Figure 1). The majority of respondents in all regions reported seeing 15 or less follow-up TS patients/month; 5% of CA and EU respondents reported seeing greater than 30 follow-up TS patients/month (Supplementary Figure 2). Respondents from the US reported seeing the fewest number of both new and follow-up TS patients/month.

The most commonly seen age range for TS patients was 6–11, regardless of region, closely followed by ages 12–18. Approximately a third of respondents in the UK and EU reported seeing adult TS patients (>18 years old), whereas just under a quarter of clinicians in CA and the US reported doing so (Supplementary Figure 3).

**Figure 2:**
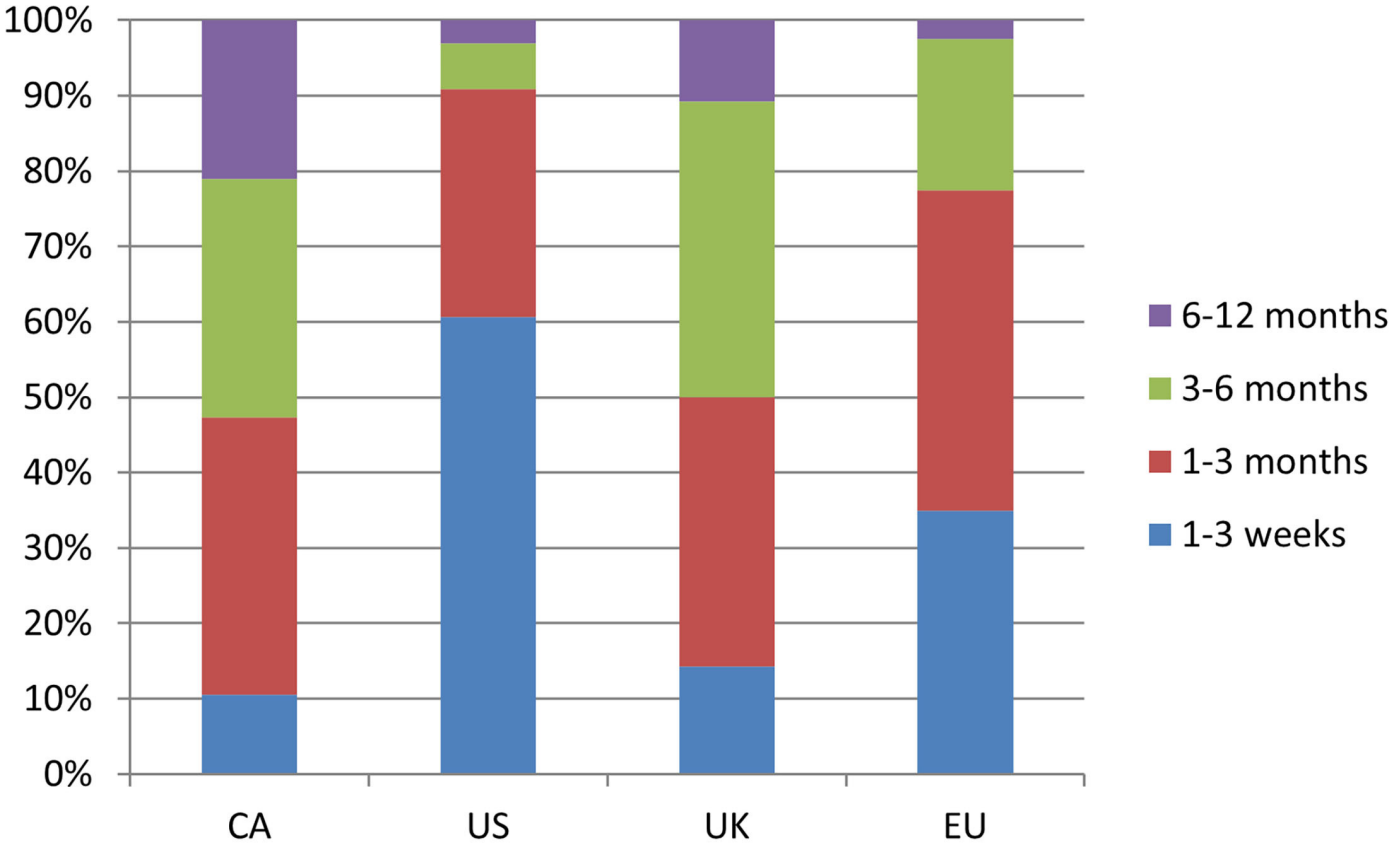
Response break-down for the question: “How long is the wait time on average for new patients with TS?”

In terms of health services delivery management, the large majority of respondents, regardless of region, reported that patients are booked in the order referrals are received and are not commonly triaged based on severity or urgency (Supplementary Table 1). The most common average wait time for new TS patients to be seen by a clinician varied greatly by region; 1–3 weeks for the US (61%), 1–3 months for CA (37%) and the EU (42%), with the UK having the longest wait time at 3–6 months (39%) (Figure 2). Survey participants were also asked about the availability of telemedicine services, that is, the use of telecommunication and information technology to provide clinical health care services from a distance. Only in the US were telemedicine services commonly available (84%), whereas access to such services was less common in CA (42%), the UK (21%) and the EU (8%) (Supplementary Table 1).

**Figure 3:**
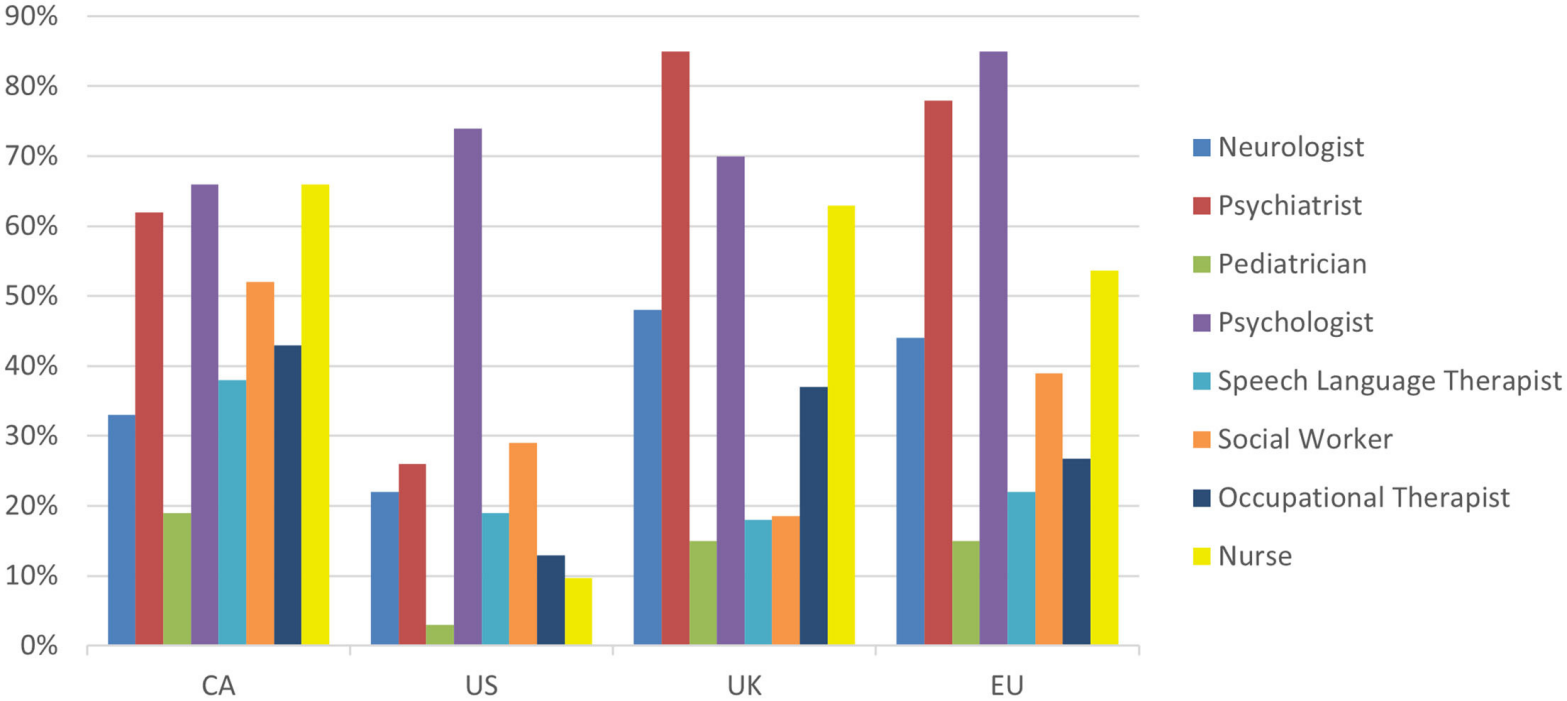
Response break-down for the question: “Who are the healthcare providers of your clinical team?”

Many respondents in the EU, UK, and CA, reported having a diverse interdisciplinary team, often consisting of psychiatrists, psychologists and nurses, with neurologists, social workers and occupational therapists being far less common, especially in the US (Figure 3). There were some common trends in clinical resource management between the geographical regions (see Supplementary Table 1). More than 70% of clinicians in all regions reported using intake questionnaires. Additionally, the average initial consultation length for the vast majority of clinicians was reported as being between 1–2 h, with patients subsequently being followed for as long as needed (Supplementary Figure 4). Most respondents prioritized clinical time and resources to provide comprehensive and continuing treatment to existing patients rather than assessing as many new patients as possible. More than half of all respondents reported tracking patient outcomes, for example, by using quality of life, patient satisfaction or symptom-based measures (Supplementary Table 1).

### Clinical Management

Survey participants were asked to indicate which TS comorbidities they manage (Supplementary Figures 5, 6). The vast majority of respondents reported managing obsessive-compulsive disorder (OCD), attention-deficit/hyperactivity disorder (ADHD) and anxiety disorders. Most respondents reported managing anger/rage problems, ranging from 52% of US respondents to 87% of EU respondents. As well, nearly 60% of respondents across all regions reported managing skin picking/self-injurious behaviors and sleep disorders with one exception being that only 40% of CA respondents reported managing sleep disorders. Regional differences were also found with autism spectrum disorder (ASD) management; <30% of CA and US respondents reported managing comorbid ASD, compared to the UK and EU in which more than 50% reported doing so.

**Figure 4:**
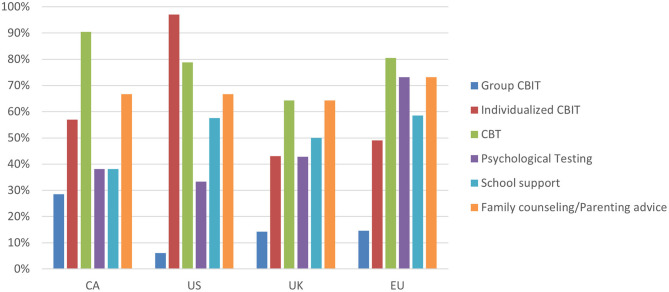
Response break-down for the question: “What services are available for TS patients at your clinic?”

In terms of TS management, respondents were asked about the therapeutic services offered at their clinic (Figure 4). Individual CBIT therapies were highest among US respondents (91%), when compared with the other regions (43–57%). As well, the majority of respondents in all regions reported offering psychoeducation at their clinic, ranging from 68% of UK respondents to 83% of EU respondents.

When looking at order of intervention preferences among the respondents, only physician responses were included since in most regions, only physicians are licensed to prescribe pharmacological treatments. Physician responses were then grouped by region: UK (*n* = 17), EU (*n* = 26), CA (*n* = 7) and US (*n* = 0).

Psychoeducation was the first-line choice of intervention for 57% of CA physicians and three-quarters of physicians in the EU and UK (Supplementary Figure 7). Behavioral interventions were the most popular second-line treatment choice for physicians in all regions—CA (57%), UK (59%), and EU (81%). For third-line treatment choice, pharmacological interventions were the most common choice in each region; CA (57%), UK (71%), EU (84%).

For the **first-line** medications for tics, 56% of respondents in the UK chose DRAs, and a further 38% chose A2AAs. A similar pattern was observed in the EU with 61% and 27% choosing these options, respectively. Notably the proportion was reversed in Canada, where 86% of CA clinicians chose A2AAs, while none chose antidopaminergic medications as their first-line psychotropic choice (Table 1).

**Table 1:** Percentages of first, second and third line pharmacotherapeutic preferences of physicians by region.

	**Canada (*****n*** **=** **7)**			**UK (*****n*** **=** **17)**			**EU (*****n*** **=** **26)**		
	**1^**st**^ line**	**2^**nd**^ line**	**3^**rd**^ line**	**1^**st**^ line**	**2^**nd**^ line**	**3^**rd**^ line**	**1^**st**^ line**	**2^**nd**^ line**	**3^**rd**^ line**
α-2 adrenergic agonists	85.7	14.3	0.0	35.3	17.6	5.9	26.9	23.1	19.2
Dopamine antagonists	0.0	71.4	0.0	58.8	35.3	0.0	61.5	19.2	7.7
Tetrabenazine	0.0	14.3	14.3	0.0	0.0	17.6	3.8	3.8	19.2
Stimulants	0.0	0.0	14.3	0.0	0.0	5.9	3.8	11.5	7.7
SSRIs	14.3	0.0	0.0	5.9	5.9	11.8	0.0	15.4	7.7
Other	0.0	0.0	71.4	0.0	41.2	58.8	3.8	26.9	38.5

Dopamine antagonists were the most popular **second-line** agents for physicians in the UK (31%) and CA (71%), whereas A2AAs were the most popular in the EU (23%). Multiple physicians in the EU chose selective serotonin reuptake inhibitors (SSRIs) (15%), stimulants (12%), and anticonvulsants (8%) as their second-line agent of preference.

The most popular **third-line** treatment in CA was tetrabenazine (14%). Popular choices for third-line treatment in the UK included tetrabenazine (19%), botulism toxin (13%), anticonvulsants (13%) and SSRIs (13%). Similarly, there was a wide range of preferences in the EU with the most popular third-line choices as follows: A2AAs (19%), tetrabenazine (19%), cannabis (8%), benzodiazepines (8%), stimulants (8%) and SSRIs (8%) (Table 1).

### Tic Specialists

The regional breakdown for tic specialists, that is, those whose practice included more than 60% of patients with tics, were as follows: CA *n* = 5, US *n* = 2, UK *n* = 8, EU *n* = 7; responses were combined (*n* = 22) for descriptive reporting purposes. The majority of tic specialists (59%) reported having wait times of 3 months or longer for new patients, with wait-list management based on the order referrals are received (73%) and telemedicine services offered by the minority of respondents (41%). As well, the majority of tic specialists reported that they use intake questionnaires and track patient outcomes (73 and 82%, respectively). Greater than 70% of tic specialists reported managing comorbid OCD, anxiety, ADHD and anger/rage issues while approximately 40% or less manage comorbid ASD, learning disabilities and sleep disorders. When asked about the services available for TS patients at their clinic, 32% reported the availability of group CBIT while 73% reported the availability of individual CBIT services. Additionally, the majority (64–68%) of tic specialists reported the availability of cognitive behavioral therapy, family counseling, psychological testing and school support services at their clinic.

## Discussion

This study investigated the patterns of health care delivery to patients with TS in different geographic regions. The results of the study suggest that access to services varies greatly and finding timely professional help may be challenging for TS patients in some regions. Nearly all respondents practice in urban settings, potentially limiting access to care for TS patients in rural areas. Furthermore, only a small number of respondents in each region reported having a clinic population in which the majority of their patients have a tic disorder, indicating that there is a lack of dedicated TS clinics. Clinicians, regardless of region, reported seeing more follow-up patients than new patients, with respondents in Europe generally seeing more patients than their counterparts in other regions. Most respondents prioritized clinical resources to providing care to existing patients thus reducing the availability of services for new patients. These findings raise questions about ease of access to care for new TS patients, especially those living outside of large urban centers. With the exception of the US, most new TS patients have to wait 1 month or longer to be seen by a clinician. Difficulties in accessing services for TS patients have also been reported in previous studies from Canada (23), the US (24), Spain (25) and Australia (19), with patient respondents noting a long and difficult process before they receive a TS diagnosis (26) and a lack of knowledge regarding tics among health care specialists (25).

In general, tic specialists reported long wait times for initial consultations. The combination of longer wait times and fewer specialists is such that most patients seek treatment from non-specialized physicians and/or non-physician clinicians. Shortages of experts and knowledge gaps may explain some of the reasons why access to services is limited for TS patients (27–29). Additionally, there is a lack of multidisciplinary teams with expertise to manage TS and its co-morbidities (19, 28) which may be especially relevant to the US, as only a few US respondents reported the availability of such teams in comparison to the other regions. The Tourette Association of America (TAA) recognized this problem a few years ago and encouraged the development of multidisciplinary clinics by offering their Center of Excellence designation to select clinics in the US. The benefits of such interprofessional teams allow for collaborative treatment, pooled expertise and often, shorter wait-times. A feasible solution may be an interprofessional care model (30) where expert health care providers assess a patient and develop a treatment plan that is then implemented by local, non-expert health care providers, with the experts providing ongoing support via communication and re-assessment as required.

The most common age range of TS patients seen by our respondents was 6–11, followed by 12–18, regardless of region. The predominance of child and youth patients is understandable as TS peaks in early puberty and wanes during the late teens, with only a subset of TS patients exhibiting symptoms that require services into adulthood (31, 32). Although only a minority of adults continue to exhibit significant tics, these are often impairing. Our data indicates that adults with TS are even more underserviced than children and adolescents. However, regional differences were apparent regarding services for adult TS patients, with the EU and UK seeing more adult patients than the respondents from North America. These results may also reflect the possibility that many of the health care providers who responded to our survey focus solely on pediatric populations, however this was not directly assessed.

Psychoeducation was reported as the first-line intervention for all the regions and is offered by the majority of all regional respondents. Psychoeducation is crucial in addressing the stigma of TS, understanding the course of the disorder, and teaching acceptance and coping strategies to patients and their families (33–35). Providing TS education and coping mechanisms may be sufficient in reducing tic-related disability, as patients with mild tics often do not require additional treatment.

For patients with more severe tics who require behavioral and/or pharmacological therapies, guidelines have been published in Europe (9, 34, 36), Canada (8, 35) and the US (33, 37) to promote better integration of treatment strategies using evidence-based medicine. Most respondents in the current study indicated that after psychoeducation, behavioral therapy was their preferred treatment choice, followed by pharmacological interventions. Based on the evidence, the guidelines agree that CBIT should be the first-line treatment, even though it is resource intensive and requires a trained therapist, who are not widely available. The majority of CA and US respondents indicated that they offer individual CBIT therapy. The high rates of individual CBIT offerings in the US may speak to the success of the TAA's initiative to train clinicians in CBIT, which was funded by the Center for Disease Control. As well, the recent Treating Tourette Together summit in the US further identified increasing access to CBIT as a priority (38). Since all of our US respondents came from a list provided by the TAA, which includes practitioners who have attended their CBIT training workshops, respondent answers are unlikely to be representative of general access to CBIT for TS patients in the US, which might explain differences with other studies reporting low rates and limited access to psychotherapy in the US (26, 39, 40).

The relatively low availability of CBIT in the UK and EU may result from barriers to implementation, including lack of clinician training and the need for a series of sessions (6). CBIT delivered via telemedicine has been shown to result in similarly significant tic reductions when compared to face-to-face delivery, demonstrating the value of telemedicine services in healthcare delivery (41). With the exception of the US, the majority of all other respondents, especially in the EU and UK, indicated that telemedicine services were not available at their clinics. The implementation of telemedicine services allows for greater dissemination of healthcare services, such as CBIT, to a wider range of patients including those living in rural settings, which is especially relevant as almost all of our respondents practice in urban settings. However, for this technological development to result in increased access to CBIT, a larger number of trained clinicians will be required; additionally, issues of costs and insurance coverage for CBIT will have to be addressed (6).

There were differences among the regions with regards to first-line pharmacotherapy preferences which might reflect the influence of various TS treatment guidelines. Based on a systematic assessment of the evidence, the Canadian guidelines recommend clonidine and guanfacine as first-line pharmacotherapy for tics (8); this is reflected in our data, as the majority of CA clinicians reported prescribing A2AAs first, findings which are further supported by published Canadian TS prescribing trends in which recommendations for A2AAs increased over the study period (42). In contrast, EU and UK respondents preferred DRAs, which is consistent with the published European guidelines. The EU guidelines, driven largely by expert experience and opinion, recommend risperidone, a DRA, as the first-choice agent for the treatment of tics (9). Our results are also supported by investigations of TS pharmacotherapy trends in the UK in which the DRA aripiprazole was the most commonly prescribed medication (43), and in the EU where aripiprazole and risperidone were the most commonly prescribed medications to adult and children patients with TS, respectively (44). While there were no US physicians in the survey sample, the most recent US guidelines recommend A2AAs (33), which is supported by US studies reporting that A2AAs are the most commonly used class of medication in TS (39, 40).

It is clear that DRAs and A2AAs are the first or second choice for most physicians treating patients with TS. However, there was less consensus regarding second- and especially third-line treatment choices, with some physicians choosing SSRIs, stimulants, tetrabenazine, botulism toxin injections and benzodiazepines despite weak or absent evidence of their efficacy against tics. Specifically, there is only low quality evidence for the efficacy of agents such as tetrabenazine in treating tics, while other agents such as SSRIs and stimulants, are rarely used to treat tics on their own and can in fact exacerbate tics in some individuals (45, 46). Alternatively, such responses may have referred to treatment choices for comorbid OCD and ADHD rather than for treatment of tics, even though the survey question specifically asked about pharmacotherapeutic preferences when treating tics. Nevertheless, the current results indicate that there is little consistency in second- and third-line medication preferences and illustrates the need for better treatment options for TS patients.

Our study was not without limitations. We did not include Latin America, Asia or Africa, and while we made efforts to obtain data from Australia, we were unsuccessful. The response rates in all regions were low thus limiting the generalizability of the results, and the overall sample size was small. Many clinicians could not be reached due to outdated and/or incorrect contact information. The questionnaire was only available in English, which made it difficult to get results from non-English speaking clinicians. As well, we reached out only to the contacts that were provided by individual TS associations. This may have biased the sample as the practices reported in our survey may not be representative of the care received by TS patients who see health care professionals not listed on the TS associations' contact lists. For example, family physicians who provide primary care for their patients with TS are likely under-represented in TS association listings. Additionally, the US sample did not include any physicians and thus could not be included in the treatment preference comparisons. However, we believe that the responses collected from the US non-physician clinicians provide important insight about clinical care practices in the US since many of these clinicians take on critical roles in the management of tics. Finally, since our survey was conducted a few years ago, there may have been changes in some practices. For example, there has recently been a markedly accelerated implementation of telemedicine since the COVID-19 pandemic due to self-isolation measures.

TS is a treatable disorder with a good prognosis; thus, it is paramount that appropriate, evidence-based treatments are readily accessible to patients with the condition. Developing an international agreed upon treatment algorithm may be beneficial. Interprofessional care, accessibility of services, and multi-modal management approaches are needed to contribute to an ideal care facility for patients with TS (47–49). These parameters are recommended as the base of any specialized TS clinic with the goal of improving health service delivery to TS patients.

## Data Availability Statement

The raw data supporting the conclusions of this article will be made available by the authors, without undue reservation.

## Ethics Statement

Ethical review and approval was not required for the study on human participants in accordance with the local legislation and institutional requirements. Written informed consent for participation was not required for this study in accordance with the national legislation and the institutional requirements.

## Author Contributions

TB: design and execution of statistical analyses, writing of first draft of manuscript, as well as reviewing and critiquing subsequent drafts. RE: conception, organization and execution of research project, reviewing and critiquing manuscript drafts. EA-J: conception and organization of research project, reviewing and critiquing statistical analyses and manuscript drafts. PS: conception and organization of research project, reviewing and critiquing statistical analyses and manuscript drafts. All authors contributed to the article and approved the submitted version.

## Conflict of Interest

The authors declare that the research was conducted in the absence of any commercial or financial relationships that could be construed as a potential conflict of interest.
