# Supplementary material for: An International Survey of Health Care Services Available to Patients With Tourette Syndrome
Source: Front Psychiatry. 2021 Feb 26;12:621874. doi: 10.3389/fpsyt.2021.621874 (PMC7953144; doi:10.3389/fpsyt.2021.621874)
Supplement: Supplementary file 1 [file Data_Sheet_1.docx]

Supplementary Table 1. Resource use and allocation among TS clinicians

|  | |  | **CA**  *n=21*  ***n (%)*** | **US**  *n=33*  ***n (%)*** | **UK**  *n=28*  ***n (%)*** | **EU**  *n=41*  ***n (%)*** | **Total**  *n=123*  ***n (%)*** |
| --- | --- | --- | --- | --- | --- | --- | --- |
| Use of Intake Questionnaires: | | |  |  |  |  |  |
|  | *Yes* | | 19 (90) | 25 (76) | 20 (71) | 31 (76) | 95 (77) |
|  | *No* | | 2 (10) | 8 (24) | 8 (29) | 10 (24) | 28 (23) |
| Length of TS Patient Follow-up: | | |  |  |  |  |  |
|  | *As long as needed* | | 16 (76) | 28 (85) | 27 (96) | 35 (85) | 106 (86) |
|  | *For a specified period of time* | | 5 (24) | 5 (15) | 1 (4) | 6 (15) | 17 (14) |
| How are Resources Allocated: | | |  |  |  |  |  |
|  | *To provide comprehensive and continuing treatment to existing patients* | | 10 (48) | 25 (76) | 15 (54) | 21 (51) | 71 (58) |
|  | *To assess as many new patients as possible* | | 5 (24) | 0 (0) | 2 (7) | 5 (12) | 12 (10) |
|  | *Neither of the above is the predominant factor* | | 6 (28) | 8 (24) | 11 (39) | 15 (37) | 40 (32) |
| Wait-list Management: | | |  |  |  |  |  |
|  | *Triage* | | 8 (38) | 5 (15) | 8 (29) | 11 (27) | 32 (26) |
|  | *In order of referral received* | | 20 (62) | 28 (85) | 20 (71) | 30 (73) | 91 (74) |
| Availability of Telemedicine Services: | | |  |  |  |  |  |
|  | *Yes* | | 9 (43) | 28 (85) | 6 (21) | 4 (10) | 47 (38) |
|  | *No* | | 12 (57) | 5 (15) | 22 (79) | 37 (90) | 76 (62) |
| Are Patient Outcomes tracked: | | |  |  |  |  |  |
|  | *Yes* | | 11 (52) | 20 (61) | 24 (86) | 29 (71) | 84 (68) |
|  | *No* | | 10 (48) | 13 (39) | 4 (14) | 12 (29) | 39 (32) |

Supplementary Figure 1. Response break-down for the question: “On average how many new TS patients do you see per month?”

Supplementary Figure 2. Response break-down for the question: “On average how many follow-up TS patients do you see per month?”

Supplementary Figure 3. Response break-down for the question: “What age ranges are your TS patients?”

Supplementary Figure 4. Response break-down for the question: “How long is the average initial consultation length?”

Supplementary Figure 5. Response break-down for the question: “What comorbidities do you manage?”

Supplementary Figure 6. Other comorbidities managed by respondents

Supplementary Figure 7. First, second and third line therapeutic interventional preferences of physicians by region
